# Impact of Investor Behavior and Stock Market Liquidity: Evidence from China

**DOI:** 10.3390/e21111111

**Published:** 2019-11-13

**Authors:** Shulan Hu, Meiling Zhong, Yanli Cai

**Affiliations:** School of Statistics and Mathematics, Zhongnan University of Economics and Law, Wuhan 430073, China; m18813092109@163.com (M.Z.); caiylzuel@163.com (Y.C.)

**Keywords:** investor behavior, market liquidity, margin trading and short selling, entropy-weight method

## Abstract

Investor behavior is one of the important factors that affects market liquidity. It is very interesting to find out how investor behavior affects stock market liquidity. The Investor sentiment changes and information cognitive ability affect not only their expected returns but also market liquidity through short-selling restrained market behavior. This paper gives a comprehensive index of investor sentiment based on the entropy method. According to the empirical analysis based on evidence from China, we obtain the following results: The investor sentiment has a positive impact on market liquidity; the development of margin trading has curbed the positive impact of investor sentiment on market liquidity; the information cognitive ability has a negative impact on market liquidity; the explosive information volume enhances the market liquidity in the bull, weakens the market liquidity in the bear, and has no significant impact while shocked.

## 1. Introduction

Market liquidity interacts with expected market returns and price fluctuations. Healthy market liquidity is the premise of a healthy and orderly development of the securities market. There are some methods to estimate market liquidity. For example, Kyle [1] used tightness, depth and resiliency; Ouyang [2] based it on the transaction cost, commission quantity, equilibrium price, and market shock; and Chung [3] used the hierarchy of liquidity and uncertainty. Investor behavior is the performance of investors in the market-trading process, the basis of market transactions, and one of the important factors affecting market liquidity.

Traditional financial-market theory (see [4]) often assumes that every investor is a rational person, but this is often hard to be satisfied in reality. Behavioral finance theory shows that most investors are actually behavioral investors, and their investment behavior is not always rational. Behavioral finance theory studies the influence of investor sentiment, information cognitive ability, and expected returns on investment behaviors. It was found that the sentiment change of irrational investors is an important factor affecting stock mispricing. In China’s A-share market, individual investors account for a large proportion, mostly lacking professionalism, showing speculative or “herd behavior”, or over- or under-reaction (see [5]).

Research on investor behavior and market liquidity mainly includes (1) analyzing factors affecting investor behavior and their impact on financial markets from the perspective of behavioral finance. Derrien [6] and Ljungqvist et al. [7] found that investor sentiment had an impact on the high receipts of the IPO on the first day and the long-term low return of the stock; Verma et al. [8] proposed that investor sentiment has an asymmetric spillover effect on the stock market, and the spillover effects of both bullish and bearish sentiment are different; Barberis et al. [9] pointed out that investor behavior is affected by the level of information cognition, for example, investors’ “conservative bias” and “selectivity bias” cause investors to “underreact” or “overreact” to market information. (2) A series of studies were carried out on the interpretation and measurement of market liquidity. Market liquidity reflects the market’s liquidity and operational efficiency. Amihud [10] constructed a reverse indicator of market liquidity to measure the depth of financial markets and establish a link between market liquidity and expected market returns; Goyenko et al. [11] compared various market-liquidity measurement indicators, analyzed the effectiveness of each method on market liquidity, and finally pointed out that Amihud’s illiquidity indicators better reflected the impact of price on liquidity; Huang and Yang [12] revised the Amihud indicator from the characteristics of China’s securities market and constructed a new indicator to characterize the impact of command flow on price; Yang and Huang [13], and Liu and Zhu [14] used this indicator to conduct research on the Chinese securities market. (3) A series of studies on the correlation between investor sentiment and market liquidity. Liu [15] proposed Granger causality between investor sentiment and market liquidity; Yao [16] analyzed the existence of two-way Granger causality between investor sentiment and market liquidity, and the effect between them was asymmetrical; Yang and Zhou [17] pointed out that investor sentiment causes a sudden change in stock market yield and asset-price plunging by affecting investor trading behavior, which, in turn, affects the stability of financial markets.

From the aspect of what affects liquidity on the market, we can find some works on factors such as noise trading [18,19,20], investor overconfidence [21], margin trading and short selling [22,23]. Debata et al. [24] found a positive (negative) effect of investor sentiment on liquidity (illiquidity). Statman et al. [21] showed that investors overconfident about their valuation and trading skills can explain high observed trading volume. Wang and Zhao [23] studied the impact of margin trading and short selling on China’s stock market. Zhang et al. [22] proposed that margin trading and short selling can promote stock market liquidity. Yang et al. [25] studied the influence of investor structure and buying and selling behavior on market liquidity. There are also a few studies on the relationship between investor behavior and market liquidity. Baker and Stein [18], and Baker and Wurgler [26] considered investor sentiment, market liquidity, and stock returns in a research framework, but without discussing how investor behavior affects market liquidity; Liu et al. [14] analyzed the influence of investor behavior on market liquidity from investor sentiment, information cognition, and short-selling constraints.

In the paper, we consider the influencing factors of investor behavior, including: Investor sentiment, investor information cognition, and margin trading and short selling in the indicator system. Besides, we analyze the interaction among investor sentiment, investor information cognitive ability, margin trading, short-selling restrain variables, and investor sentiment short-selling restraints, and their impact on market liquidity. Our main objectives were to (1) introduce the Baidu Index as a proxy variable for investor sentiment (see [27,28,29,30]), (2) and use the entropy-weight method to construct a more comprehensive index to represent the investor sentiment of the whole market. The Baidu index can reflect the amount of explosive information and present the impact of explosive information on market liquidity in different markets.

The paper consists of five parts. In Section 1, we give the background, objective, and frame of the paper. In Section 2, we analyze the types of investors for constructing investor-sentiment indicators in the stock market. In Section 3, we consider the measurement of the research objects to build the index system. In Section 4, we construct the empirical equation and analyze the impact of each variable on market liquidity. In the last section, we summarize the research results and propose suggestions.

## 2. Investor Sentiment

According to the results from [14,31], there are three types of investors in the stock market: Informed, rational, and noise investors. The informed investors are not easily influenced by external information, and they constitute a small proportion of the market, and consequently, they have little influence on market liquidity. Rational investors will not be blindly affected by external information, nor will they easily spread the influence, making their impact on market liquidity very small. In the long run, noise traders dominate the whole market, thus making market efficiency disappear ([19]). Their expectations of the market are vulnerable to the external environment and frequently change, so these lead to changes on market liquidity.

### 2.1. Basic Proxy Variable

Investor sentiment directly affects the subjective judgment of investors on the expected return of stocks, which, in turn, affects their investment behavior and the trading market. Investors are susceptible to subjective awareness when making investment decisions, so stocks cannot be rationally valued and systematic deviations happen between their expected returns and real values. However, investor sentiment is well-recognized but difficult to measure. In early research [32,33,34], survey data and proxy variables of market transactions were often used to represent it. Commonly used basic proxy variables (see [35,36,37,38,39]) can be divided into two main categories: Single variables and comprehensive indicators. We constructed a comprehensive index of investor sentiment with the entropy-weight method in order to more fully reflect investor sentiment in the market.

The method of determining the underlying proxy variables is to first construct investor-sentiment indicators from the micro- and macroperspective of behavioral finance, and, second, estimate limited investor attention as a basic proxy variable.

With the rapid development of information networks, people are increasingly accessing information on the stock market through the Internet. Due to time and energy constraints, investors are hard to acquire and analyze all the related information, then, they cannot adjust their investment behavior in time. Therefore, the concept of investor limited attention is put forward. Wang [40] showed that the irregular fluctuation of limited attention can reflect investors’ sentiment and the change of attention at the same time, and it is an important index to describe investor behavior. Zhou & Huang [41] added investor’s limited attention into the construction of comprehensive sentiment index, and proposed that limited attention index can well represent investor’s sentiment. Therefore, we introduce limited attention index as a measurement of investor sentiment.

In the current research, investor limited attention are mostly measured by search-volume data in the search engine ([29,30]) and number of posts on the website ([42,43]) and so on. Considering the availability of data, we use Baidu Index as the proxy variable of investor sentiment. “Baidu” is the largest search engine in China, and Baidu Index is a statistical index constructed by the search volume of users to measure the search situation of users on Baidu search engine for a period of time.

### 2.2. Entropy-Weight Method

The entropy-weight method ([44]) is an objective method to determine factor weight. The influence of each factor on comprehensive evaluation is judged by the degree of dispersion of the factor.

The steps of the entropy-weight method are as follows:

Step 1. Standardization 

Suppose the original data are $X=(x_{1},x_{2},\ldots ,x_{k})$, where factor $x_{i}$ is an *n*-dimensional column vector. Because the measurement units of each indicator are different, data should be standardized first.

(1) $$y_{ij}=\frac{x_{ij}-\min\left(x_{.j}\right)}{\max\left(x_{.j}\right)-\min\left(x_{.j}\right)},\;\;i=1,2,\ldots ,n;\;j=1,\ldots ,k.$$

Step 2. Information entropy 

Calculate the information entropy of each factor according to the definition of information entropy in information theory, which is given by:

(2) $$E_{j}=-\frac{1}{\ln(n)}\sum_{i=1}^{n}p_{ij}\ln p_{ij},\;j=1,2,\ldots ,k.$$

In the formula, $P_{ij}=\frac{y_{ij}}{\sum_{i=1}^{n}y_{ij}}$, when $P_{ij}=0$, define $\lim_{P_{ij}\to 0+}P_{ij}\ln P_{ij}=0$.

Step 3. Weight 

Calculate the corresponding weights according to the information entropy of each factor:

(3) $$w_{j}=\frac{1-E_{j}}{k-\sum_{j=1}^{k}E_{j}},\;j=1,2,\ldots ,k.$$

Step 4. Comprehensive indicator 

Based on the weight values, integrate all factors to construct a comprehensive index as follows:

(4) $$S=\sum_{j=1}^{k}w_{j}x_{j}.$$

## 3. Index System

### 3.1. Market Liquidity

Liquidity can be used to observe the structure and operation of the market. A market with good liquidity can realize its price-discovery function and complete the optimal allocation of resources, so that the securities market can develop healthily and in an orderly manner.

Among many ways to measure market liquidity, illiquidity proposed by Amihud [10] is widely used. Based on this index, we build a reverse indicator of market liquidity:

(5) $$Illiq_{t}=\frac{1}{Day_{t}}\sum_{i=1}^{Day_{t}}\frac{\left|R_{ti}\right|\times N_{ti}}{Tvalue_{ti}}\times 10^{10}.$$

As we all know, the illiquidity proposed by Amihud means the mean of the ratio between the weekly return and average turn-over of a stock. Here, we add $N_{ti}$ to eliminate the influence of the increase of circulating stock on the turnover, adding $10^{10}$ to adjust the Illiquidity index value to an appropriate level. $N_{ti}$ stands for the number of effective stocks on the *i*-th trading day of the *t*-th week. $R_{ti}$ stands for the return rate of the A-share market on the *i*-th trading day of the *t*-th week, which is directly available from the China stock market and accounting research (CSMAR) database. $Tvalue_{ti}$ stands for the turnover of the *i*-th trading day of the *t*-th week, and $Day_{t}$ stands for the number of trading days in the *t*-th week.

### 3.2. Investor Sentiment

Behavioral finance believes that changes in the market can reflect the current investor sentiment in a timely manner. We divided all investors into internal and external market investors, and constructed internal and external investor-sentiment indicators based on market changes.

Internal market sentiment indicators: Taking the change rate of CSI 300 Index as a proxy variable, the changes in the CSI 300 Index can reflect the sentiment of investors within the market. $Senti1_{t}$ equals to $\frac{P_{t}-P_{t-1}}{P_{t-1}}$, $P_{t}$ means the closing price of CSI 300 on the last trading day of the *t*-th week. The CSI 300 is a capitalization-weighted stock market index designed to replicate the performance of the top 300 stocks traded in the Shanghai and Shenzhen stock exchanges. The index is compiled by the China Securities Index Company, Ltd. Its increase or decrease directly affects the investment sentiment of investors within the market.

External market-sentiment indicators is the number of relatively new accounts in the A-share market, which is given by:

(6) $$Senti2_{t}=\frac{Newac_{t}}{avg(Newac)}.$$

$Newac_{t}$ stands for the number of new accounts of the A-share stock in the *t*-th week, and avg(Newac) stands for the average of the new A-share accounts of each week over the entire sample period.

According to the research of Yu and Zhang [29], the Baidu index of CSI 300 was used to build an indicator of investor sentiment. It was constructed based on the weekly search-volume data of the Baidu search engine, reflecting the number of times that a keyword was searched in the Baidu engine. Senti3 is given by:

(7) $$Senti3_{t}=\ln\left(NameIndex_{t}+NumberIndex_{t}\right).$$

$NameIndex_{t}$ and $NumberIndex_{t}$ stand for the Baidu index value of the stock index and the stock code of the CSI 300 Index in the *t* week, respectively.

According to the entropy-weight method, the weight values of basic variables are $w_{1},w_{2},w_{3}$, and the basic proxy variables were weighted to construct a comprehensive indicator of investor sentiment:

(8) $$Senti_{t}=w_{1}Senti1_{t}+w_{2}Senti2_{t}+w_{3}Senti3_{t}.$$

### 3.3. Information Cognitive Ability

The speed with which investor behavior changes with new information in the stock market depends on the ability to recognize new information. An investor’s cognitive-ability indicator is constructed as follows:

(9) $$Congi_{t}=\frac{1}{Day_{t}}\sum_{i=1}^{Day_{t}}\frac{\left|Tvolume_{t(i+1)}-Tvolume_{ti}\right|}{(Tvolume_{t(i+1)}+Tvolume_{ti})/2}.$$

$Tvolume_{ti}$ represents the trading volume of the stock on the *i*-th trading day of the *t*-th week. The first trading day of the week is the next trading day after the last trading day of the previous week. $\left|Tvolume_{t(i+1)}-Tvolume_{ti}\right|$ stands for the absolute value of the difference between the two trading days. The larger the value is, the lower the correlation between the trading volume of the two adjacent trading days, and the higher the investor information cognitive ability.

### 3.4. Margin Trading

China’s securities market has always lacked a short-selling mechanism. The stock market is a unilateral market and cannot be sold short. On 31 March 2010, the pilot of margin trading and short-selling transactions was opened. Investors could make short sales by borrowing funds or underlying stocks from other brokers. It was the beginning of short-selling transactions in China’s securities market. There were many controversies about the nature and role of short selling, but a large number of studies confirmed that short-selling restrains have an influence on the stock market. In order to study the effects of short-selling restrains and investor sentiment on stock market liquidity, we developed short-selling indicators in this paper through the development of margin-trading and short-selling businesses.

The margin-trading and short-selling business experienced five expansions since its official launch on 31 March 2010, of which the expansion time was 5 December 2011 (48th week), 31 January 2013 (105th week), 16 September 2013 (137th week), 22 September 2014 (189th week), and 12 December 2016 (303th week). We set dummy variables $Short_{1}∼Short_{5}$ with these weeks as the turning point, for example, for the sample earlier than 5 December 2011, $Short_{1}$ is 0; otherwise, it is 1.

### 3.5. Stock Market

Rao and Tu [45] proposed that there is asymmetric influence between investor sentiment and stock market returns under different stock market conditions. The investor-sentiment indicator actually reflects the explosive-information volume of network information. Furthermore, we studied the impact of explosive information on market liquidity by analyzing the relationship between investor sentiment and market liquidity under different stock market conditions.

There stock market can be divided into the bull, bear, and shock market. Here, we divided it based on the divergence pattern of the long-term average, which has three forms: Upward divergence, downward divergence, and average entanglement, which correspond to the bull, bear, and shock markets. Long-term moving averages commonly used in the market are: The 60-, 120-, and 250-day lines. In weekly data, 12-, 24-, and 50-week lines are used instead. We set dummy variables Hang1∼Hang3, which stand for bull, bear, and shock market, respectively. For example, when the moving averages show up as diverging, the stock market behaves as a bull market, at which time Hang1=1; otherwise, 0.

## 4. Analysis

### 4.1. Data Sources

China’s stock market includes the A-share market, B-share market, and the GEM market. The A-share market is the main representative of the situation of China’s stock market. We took the comprehensive A-share market (excluding the GEM) as the research object. Weekly data from 4 January 2011 to 3 June 2019 were selected, excluding missing data, data on 3 June 2019, and the outlier. There were 415 samples in all. The data came from CSMAR and Wind. In this paper, we constructed a model from 393 weeks, from 4 January 2011 to 24 December 2018, and used the remaining 22 weeks to test the validity of the model.

### 4.2. Investor-Sentiment Index

We constructed three basic proxy variables of investor sentiment. Senti1 increased as the market improves, Senti2 became large with new investors in the market. Senti3 became large with investor sentiment, stock search volume, and the Baidu index. So, the larger the indicator value is, the higher the investor sentiment.

Let $x_{1}=Senti1,\;x_{2}=Senti2,\;x_{3}=Senti3$, and the matrix of sample data can be defined as follows:

(10) $$X=\left(x_{1},x_{2},x_{3}\right)=\left(\begin{array}{ccc} x_{11} & x_{12} & x_{13}\\ \vdots & \vdots & \vdots \\ x_{m1} & x_{m2} & x_{m3} \end{array}\right)$$

where sample size is m=393. According to the entropy-weight method introduced above, the entropy values of the basic proxy variables and their weights were as Table 1:

The investor-sentiment indicator can be calculated as follows:

(11) Senti=0.231Senti1+0.233Senti2+0.536Senti3

**Figure 1:**
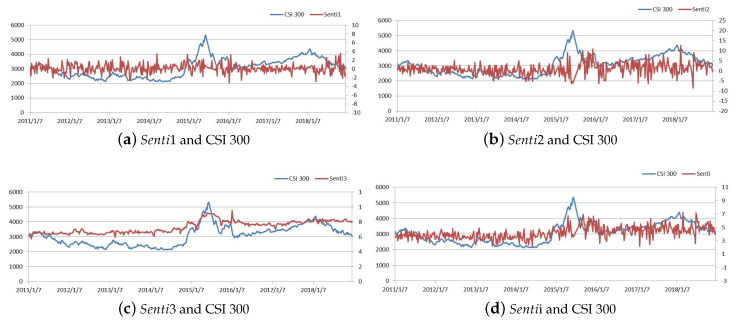
Different Investor sentiment indicators and the CSI 300 index.

Why build a comprehensive indicator? Can the indicator truly and effectively reflect the general sentiment of investors? The comparisons of different investor sentiment indicators and the CSI 300 index during the sample period are in Figure 1:

Figure 1a shows that investor sentiment does not fluctuate with the CSI 300; Figure 1b shows that the volatility of external investor sentiment is basically consistent with fluctuations of the CSI 300, but the trend does not match; Figure 1c shows that investor sentiment from effective investor attention is similar to that of the CSI 300 index. Based on these characteristics, we constructed a comprehensive investor sentiment to combine all advantages for the stock market in Figure 1d.

**Figure 2:**
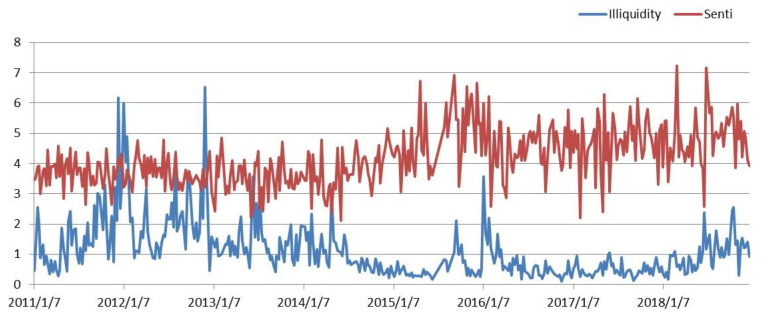
Market illiquidity and investor sentiment.

Before the empirical study, the relationship between investor sentiment and market liquidity was visually observed through line graphs. Figure 2 shows that the decrease in market illiquidity is accompanied by higher investor sentiment, and the increase of illiquidity is followed by lower investor sentiment. There may be a negative relationship between investor sentiment and market illiquidity.

### 4.3. Analysis of Research Results

We studied the effects of investor sentiment, information cognition, short-selling restrains, and their cross-variables on market liquidity. There are many factors that affect market liquidity: Mishkin [46] indicated that an increase in interest rates and in uncertainty about the future direction of government policies promotes financial crises. Li and Peng [47] proposed that investor sentiment and stock market liquidity are Granger reasons for economic growth. Ding [48] showed that an M2 year-on-year growth rate had significant impact on the liquidity of China’s bond market. Therefore, we considered the following control variables: Riskfree interest rate, economic uncertainty index, CPI, economic sentiment index, and M2 year-on-year growth rate. All data came from the Wind database. The ADF test found that these variables were nonstationary, so we smoothed them with the methods of first-order difference, and named them rate, uncertainty, cpi, esindex, and m2, respectively. In addition, Yao [16] showed two-way causality between investor sentiment and market liquidity. To prevent endogenous effects on the model results, hysteresis variable $Illiq_{t-1}$ was added, and variable Senti was standardized. The model was as follows:

(12) $$\begin{array}{cc} Illiq_{t}=\beta_{0}+\beta_{1}Senti_{t}+\beta_{2}Cogni_{t}+ & \beta_{3}Short_{t}\times Senti_{t}+\beta_{4}rate_{t}+\beta_{5}uncertainty_{t}\\ & +\beta_{6}cpi_{t}+\beta_{7}esindex_{t}+\beta_{8}m2_{t}+\beta_{9}Illiq_{t-1}+\varepsilon_{t} \end{array}$$

The results showed that only two control variables were significant, cpi and esindex. It was found by the Ljung–Box test that there was autocorrelation of the residual sequence. Wooldridge pointed out the sequence correlation of the error of the dynamic model, usually due to the regression function not being completely set, and the hysteresis variable could be added at this time. Therefore, we modified the model to the following form:

(13) $$\begin{array}{cc} Illiq_{t}=\beta_{0}+\beta_{1}Senti_{t}+\beta_{2}Cogni_{t}+ & \beta_{3}Short_{t}\times Senti_{t}+\beta_{4}cpi_{t}+\beta_{5}esindex_{t}\\ & +\beta_{6}Illiq_{t-1}+\beta_{7}Illiq_{t-2}+\beta_{8}Illiq_{t-3}+\varepsilon_{t} \end{array}$$

Before the study, we performed an augmented Dickey–Fuller test (ADF for short) on the variables in the model. The results were shown in Table 2:

The stationarity test of all variables was accepted. When we added Short1×Senti or Short2×Senti into the model, the model had multicollinearity. Therefore, the model was fitted through the sample data, and the results were shown in Table 3:

In these models, the explanatory variables were independent because VIF values of the variables were less than 10, and the Ljung–Box test and ARCH-LM test showed that Residuals do not have autocorrelation, ARCH effect.

Model 1 shows that the coefficient of Senti was significantly negative and the increase of investor sentiment enhanced market liquidity. The partial regression coefficient of Congi to Illiq was significantly positive, implying that the faster the information perception is, the weaker the market liquidity. This is because perception of new information by noise investors is mostly insufficient in China’s stock market. At that time, high investor sentiment helped to promote transactions, and the improvement of investor information’s cognitive ability weakened stock market liquidity.

The margin-trading and short-selling business indicated that investors could borrow or sell short-term or short-selling transactions from other brokers, which directly affected the trading behavior of investors. In addition, investor-sentiment changes and information cognitive ability affect their expected returns and market liquidity through short-selling-restrained market behavior.

The results of Models 1 and 4 are similar, and we believed it was unlikely that the margin-trading and short-selling business had an impact on the relationship between investor sentiment and market liquidity.

Models 2, 3, and 5 show that the coefficient of Short×Senti in these models was significantly positive, contrary to the effect of Senti on iliquidity, which indicates that the margin-trading and short-selling business inhibits the positive impact of investor sentiment on market liquidity.

We needed to test the robustness and validity of the model. A different illiquidity measurement was used to test the robustness of the results. Here, we chose CSI 300 as the object, and the following indicators based on Huang and Yang [12]. The price amplitude on the *i*-th trading day of the *t*-th week is:

$$SW_{i}=\frac{P_{i}^{max}-P_{i}^{min}}{P_{i}^{open}},$$

where $P_{i}^{open}$ is the open price of the day, $P_{i}^{max}$ is the highest price of the day, $P_{i}^{min}$ is the lowest price of the day, $V_{i}$ is the trading price on the *i*-th trading day of the *t*-th week, $D_{t}$ is the number of effective trading day for the *t*-th week. If the price amplitude on the trading day is not zero, then it is a valid trading day. Then:

(14) $$Illiq_{t}=\frac{1}{D_{t}}\sum_{i=1}^{D_{t}}\frac{SW_{i}}{V_{i}}\times 10^{7}$$

The probability of the ADF test for this Illiq was 0.043, so it was stationary. We used the same control variables as above. When we only introduced $Illiq_{t}-1$, if the residual had no autocorrelation, no higher-order lags were introduced. Short1×Senti could cause severe multicollinearity. After fitting the model with ordinary least squares, the residual had an ARCH effect; therefore, the ARCH equation must be constructed to eliminate conditional heteroscedasticity [49]. The model results were in Table 4, and there was no multicollinearity, residual autocorrelation, and conditional heteroscedasticity in these models.

The variable relationship reflected by the parameter results was consistent with Models 1–5. Investor sentiment had positive impact on market liquidity, while information cognitive ability had negative impact on market liquidity, and the development of margin trading curbed the positive impact of investor sentiment on market liquidity. Therefore, the results were robust.

**Figure 3:**
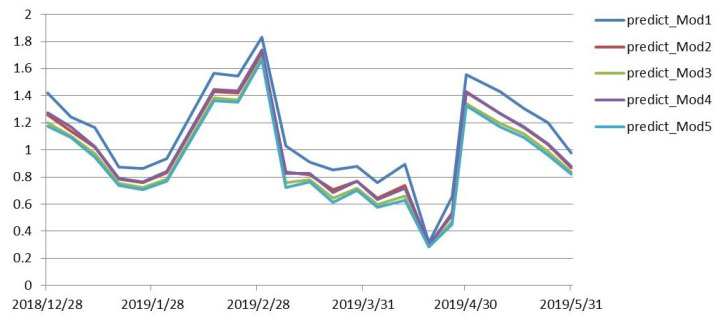
Comparison of model prediction results.

**Figure 4:**
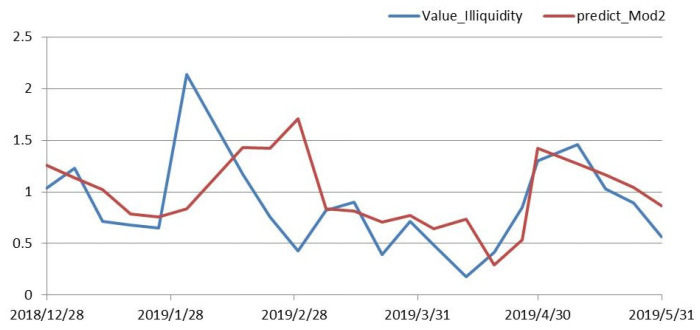
Illiquidity and its predicted value.

This was shown to determine the validity of the model, whether the predicted value of the model was consistent with the trend of the true value of illiquidity, and whether the predicted value fluctuated around the true-value data. In order to test the validity of the model, the illiquidity of the period was predicted based on market data from 24 December 2018 to 31 May 2019, and the predicted values and real-data charts were plotted as follows:

Figure 3 shows that the trend of the prediction results in the seven models was almost the same, except that the size of the predicted-value data was different. In all fitted models, Model 2 was significant with more variables. Comparing the results of Model 2 with the true value of illiquidity, Figure 4 shows that the trend of the predicted-value data was similar with the true-value trends, and the predicted values fluctuated around the true value. It is obvious that the model had a good prediction effect, and could truly reflect the relationship between variables and market liquidity.

In the above, we used Baidu Index to construct investor sentiment index. In fact, Baidu Index was built by search-volume data of Baidu search engine, which can reflect the size of network information. Here, we use Baidu Index as the proxy variable of the amount of network information to study its impact on market liquidity, Senti was standardized. The empirical equation was as follows:

(15) $$\begin{array}{cc} Illiq_{t}=\beta_{0}+\beta_{1}Senti_{t}+\beta_{2}Congi_{t}+ & \beta_{3}Hang\times Senti3_{t}+\beta_{4}cpi_{t}+\beta_{5}esindex_{t}\\ \; & +\beta_{6}Illiq_{t-1}+\beta_{7}Illiq_{t-2}+\beta_{8}Illiq_{t-3}+\varepsilon_{t} \end{array}$$

The ADF test for the cross-variables was in Table 5. Results showed that they were all stationary:

The estimated results of the model are in Table 6, and the VIF of the variables were less than 3, and the Ljung–Box test and ARCH-LM test were satisfied.

Nowadays, most people browse information from the network to grasp stock market dynamics, which affects their expectations and their investment decisions. Research shows that the coefficient is significantly negative, which indicates that explosive information volume of network information in bull market has a positive effect on stock market liquidity; the coefficient is significantly positive, indicating that there has a significant inhibitory effect in the bear market; the coefficient is not significant, meaning that there has no significant impact between the explosive information and market liquidity in the shock market. These results show that the amount of explosive information has an impact on the liquidity of the stock market, and this effect is heterogeneous. In the bull market, explosive-information volume is conducive to raising investment sentiment, thereby promoting market liquidity. In the bear market, it depresses investor sentiment and weakens market liquidity. There is no significant impact between them in the shock market.

We used a different illiquidity measurement for testing the robustness of the results. The results of the least-squares estimation had the ARCH effect, so an ARCH model was constructed. All models passed the Ljung–Box test and ARCH-LM test, and results were consistent with the above research results; the impact of explosive information on market liquidity was heterogeneous.

The empirical results in Table 7 showed that investor sentiment had a positive impact on market liquidity. Investor perception of new information has negative impact on market liquidity, which indicates that most investors in the market are characterized by a lack of awareness and tend to generate follow-up trading behavior. As investor information cognitive ability increases, it lowers market liquidity. The margin-trading and short-selling business can restrain the positive impact of investor sentiment on market liquidity, and the impact of explosive information on market liquidity is heterogeneous.

## 5. Conclusions

This paper studied the impact of investor behavior on stock market liquidity from the perspective of investor sentiment, investor information cognitive ability, and short-selling-restrained market behavior. Based on the herd mentality, inertia trading [5] and the empirical analysis of weekly transaction data of China’s A-share market, the results showed that investor sentiment had a positive impact on market liquidity and that investor information cognitive ability had a negative impact. Margin trading and short selling is the beginning of short-selling transactions in China’s securities market, which could suppress the positive impact of investor sentiment on market liquidity. The explosive amount of information also had heterogeneous impact on market liquidity, i.e., liquidity was enhanced in the bull market but was weakened in the bear market.

Most studies have only analyzed the impact of investor sentiment or margin trading and short selling on market liquidity. This paper put internal factors of investor behavior, such as investor sentiment, investor information cognition ability, and margin trading and short selling, into an indicator system to study their impact on market liquidity. We also introduced the Baidu index as a proxy variable of investor sentiment, and constructed a comprehensive index through the entropy-weight method. Furthermore, the impact of the explosive amount of information on market liquidity under different market conditions is the innovation of this paper. However, we tried to consider Senti×Congi, but there were no significant results that could be analyzed. Our further focus is to develop a more effective system or model to find out more about this relationship.

As per Liu et al. [14], in China’s A-share market, most investors’ perceptions of new information are weak, and investment sentiment is susceptible to multiple factors, which tends to cause abnormal fluctuations in the securities market. Fortunately, margin trading can inhibit the impact of investor sentiment on the market to a certain extent.

Therefore, we propose the three following suggestions: First, it is necessary to reasonably guide investor sentiment. Investor cognitive ability is weak and vulnerable to many external factors, and there is too much information in the network, so the government should appropriately guide investors’ emotions and prevent speculators to control investment sentiment that causes abnormal volatility in the market. Second, the investor education system should be improved. More and better investor education is the best solution to fundamentally solve this problem. Lastly, the margin-trading and short-selling business needs to be further developed. It can inhibit the impact of investor sentiment on market liquidity, which is of great significance to the current domestic securities market.

## Figures and Tables

**Table 1:** Entropy values and weights.

	Senti1	Senti2	Senti3
Entropy value	0.991	0.991	0.980
Weight	0.231	0.233	0.536

**Table 2:** ADF test.

Variable	*t*-Statistic	Prob
Illiq	−4.269	0.01
Senti	−3.931	0.013
Cogni	−5.009	0.01
Short1∗Senti	−3.614	0.032
Short2∗Senti	−3.468	0.045
Short3∗Senti	−3.730	0.023
Short4∗Senti	−4.982	0.01
Short5∗Senti	−4.199	0.01
cpi	−7.551	0.01
esindex	−6.037	0.01

**Table 3:** Model estimation results.

	Model 1	Model 2	Model 3	Model 4	Model 5
*C*	−0.110	−0.112	−0.129	−0.126	−0.132
Senti	−0.066 *	−0.263 **	−0.209 **	−0.102 **	−0.209 **
Congi	3.068 **	3.020 **	2.937 **	3.142 **	2.964 **
Short3×Senti		0.244 **			
Short4×Senti			0.209 **		0.197 **
Short5×Senti				0.095	0.024
cpi	0.494 **	0.507 **	0.512 **	0.496 **	0.511 **
esindex(2)	−0.201 **	−0.195 **	−0.186 **	−0.200 *	−0.187 **
AR(1)	0.383 **	0.373 **	0.379 **	0.382 **	0.379 **
AR(2)	0.204 **	0.196 **	0.196 **	0.199 **	0.195 **
AR(3)	0.146 **	0.126 **	0.136 **	0.142 **	0.136 **
$AD\;R^{2}$	0.509	0.517	0.515	0.510	0.514
*F*	58.69	53.08	52.69	51.65	46.74

Note: **, significance at 5% level; *, significance at 10% level.

**Table 4:** Model estimation results.

	Model 6	Model 7	Model 8	Model 9	Model 10	Model 11
*C*	−0.075	−0.083	−0.080	−0.090	−0.089	−0.087
Senti	−0.054 *	−0.196 **	−0.233 **	−0.179 **	−0.049	−0.178 **
Congi	2.301 **	2.275 **	2.229 **	2.194 **	2.236 **	2.173 **
Short2×Senti		0.159 *				
Short3×Senti			0.211 **			
Short4×Senti				0.173 **		0.181 **
Short5×Senti					0.022	−0.016
cpi	0.162	0.178	0.174	0.170	0.147	0.224
esindex	−0.128	−0.108	−0.111	−0.095	−0.071	−0.095
AR(1)	0.865 **	0.721 **	0.730 **	0.712 **	0.694 **	0.710 **
AR(2)		0.142 **	0.126 **	0.143 **	0.176 **	0.145 **
Variance equation.						
*C*	0.232 **	0.233 **	0.230 **	0.227 **	0.222 **	0.227 **
$RESID{\left(-1\right)}^{2}$	0.328 **	0.295 **	0.297 **	0.315 **	0.358 **	0.316 **
$AD\;R^{2}$	0.793	0.803	0.805	0.803	0.800	0.803
S.E.ofregression	0.592	0.579	0.577	0.578	0.583	0.579
SumSquaredresid	135.422	128.560	127.442	128.117	130.267	128.125
Loglikelihood	−326.332	−319.526	−317.849	−317.941	−319.899	−317.904
*D*-Wstat	2.281	2.044	2.070	2.022	1.951	2.020

Note: **, significance at 5% level; *, significance at 10% level.

**Table 5:** ADF test.

Variable	*t*-Statistic	Prob
Hang1∗Senti3	−4.199	0.01
Hang2∗Senti3	−3.730	0.023
Hang3∗Senti3	−4.982	0.01

**Table 6:** Model estimation results.

	Model 12	Mode 13	Model 14
*C*	−0.024	−0.130	−0.090
Senti	−0.059 *	−0.080 **	−0.071 **
Congi	3.045 **	3.058 **	3.079 **
Hang1×Senti3	−0.020 **		
Hang2×Senti3		0.025 **	
Hang3×Senti3			−0.008
cpi	0.484 **	0.492 **	0.497 **
esindex	−0.190 **	−0.184 **	−0.199 **
AR(1)	0.371 **	0.367 **	0.381 **
AR(2)	0.197 **	0.192 **	0.202 **
AR(3)	0.134 **	0.135 **	0.146 **
ADR2	0.514	0.516	0.509
*F*	52.34	52.82	51.38

Note: **, significance at 5% level; *, significance at 10% level.

**Table 7:** Model estimation results.

	Model 15	Model 16	Model 17
*C*	0.048	−0.085	−0.077
Senti	−0.051	−0.068 **	−0.054 *
Congi	2.283 **	2.264 **	2.300 **
Hang1×Senti3	−0.022 **		
Hang2×Senti3		0.020 **	
Hang3×Senti3			0.001
cpi	0.154	0.152	0.162
esindex	−0.112	−0.102	−0.129
AR(1)	0.830 **	0.848 **	0.864 **
Variance equation.			
*C*	0.225 **	0.222 **	0.232 **
$RESID{\left(-1\right)}^{2}$	0.336 **	0.359 **	0.327 **
$AD\;R^{2}$	0.797	0.796	0.793
S.E.ofregression	0.587	0.589	0.593
Sumsquaredresid	132.802	133.634	135.436
Loglikelihood	−321.976	−322.967	−326.321
Durbin-Watsonstat	2.243	2.286	2.279

Note: ∗∗, significance at 5% level; * significance at 10% level.
